# *Circanota*: a new genus of Sparganothini from the Neotropics, and its two new species (Lepidoptera, Tortricidae)

**DOI:** 10.3897/zookeys.462.7647

**Published:** 2014-12-10

**Authors:** John W. Brown

**Affiliations:** 1 Research Associate, National Museum of Natural History, P.O. Box 37012, Washington, DC 20013–7012, USA

**Keywords:** Costa Rica, Ecuador, morphology, Panama, *Platynota*, *Sparganothoides*, systematics

## Abstract

*Circanota*, new genus, and its two new species, *Circanota undulata* **sp. n.** (type species), from Costa Rica and Panama, and *Circanota simplex* **sp. n.**, from Panama and Ecuador, are described and illustrated. Although superficially similar to some species of *Platynota* Clemens, 1860, *Circanota* appears to be more closely related to members of a putative clade within Sparganothini defined by a slender crescent-shaped signum in the corpus bursae of the female genitalia. The most conspicuous autapomorphy for *Circanota* is the strongly undulate costa of the male and female forewing. Barcode sequence data (i.e., cytochrome oxidase I) from *Circanota undulata* (n = 12) form a tight cluster with exceedingly limited genetic divergence (less than 0.1%); specimens of *Circanota simplex* have not been sequenced. In neighbor-joining trees based on COI, *Circanota* is portrayed nearest *Sparganothoides*, which is consistent with morphological evidence.

## Introduction

Sparganothini is one of the smallest and most well-defined tribes in the family Tortricidae, with approximately 350 described species restricted almost exclusively to the New World; only a handful of species is recorded from the Palearctic (Powell and Brown 2012). The tribe has been the subject of considerable contemporary systematic work (e.g., Landry and Powell 2001, Phillips-Rodriguez and Powell 2007, Kruse and Powell 2009, Kruse 2011, Powell and Brown 2012, Brown et al. 2013, Brown 2014), and its hypothesized phylogenetic position as sister to Atteriini has been demonstrated fairly convincingly (Regier et al. 2012). Nonetheless, new species and new genera continue to be discovered, primarily from the Neotropics. The purpose of this contribution is to describe a new genus and its two new species, one from Costa Rica and Panama, the other from Panama and Ecuador.

## Methods

Dissection methods follow those presented in Brown and Powell (1991). Images of adults and genitalia were captured using a Canon EOS 40D digital SLR (Canon U.S.A., Lake Success, NY) mounted on a Visionary Digital BK Lab System (Visionary Digital, Palmyra, VA). Terminology for genitalia structures and forewing pattern elements follows Powell and Brown (2012). In descriptions of the forewing, “dorsum” refers to hind margin of the wing, i.e., the dorsal most edge of the wings when the live moth is in resting posture.

Tissue samples (i.e., one leg from a preserved adult) were used to amplify 650bp of the mitochondrial gene cytochrome oxidase 1 (COI), commonly referred to as the DNA “barcode,” using standard procedures employed at the Biodiversity Institute of Ontario, University of Guelph (e.g., Craft et al. 2010, Wilson 2012). Barcode data, along with images of the vouchers, are stored in BOLD (Barcode of Life Database).

Specimen depositories are abbreviated as follows: INBio, Instituto Nacional de Biodiversidad, Santa Domingo de Heredia, Costa Rica; MEM, Mississippi Entomological Museum, Mississippi State, MS, U.S.A.; and USNM, National Museum of Natural History, Washington, D.C., U.S.A.

## Results

### Circanota

Taxon classificationAnimaliaLepidopteraTortricidae

Brown gen. n.

http://zoobank.org/B5C859DD-C5B1-4C49-8742-FF0E93BFF688

#### Type species.

*Circanota undulata* Brown, sp. n.

#### Diagnosis.

*Circanota* is superficially most similar to *Platynota* Clemens, 1860, with a comparable forewing length and pattern, and long porrect labial palpi. *Circanota* can be distinguished from *Platynota* and all other sparganothine genera by the strongly undulate costa of the forewing in both sexes. Although females of a few species of *Platynota* (e.g., *Platynota flavendana* Clemens, 1860, *Platynota rostrana* (Walker, 1863)) have a slightly undulate costa, males typically have an evenly arched costa. Males of *Circanota* lack the complex scaling of the frons typical of many, but not all, *Platynota*, and the labial palpi of *Circanota* lack pronounced sexual dimorphism; the palpi are conspicuously longer in the female in most Neotropical *Platynota*.

The female genitalia of *Circanota* are represented by two distinct types. In the type species, *Circanota undulata* sp. n., the anterior (typically cup-shaped) part of the sterigma, possibly homologous with the antrum, is broad and asymmetrical, unique within Sparganothini. In contrast, in *Circanota simplex* the structure is unmodified and similar to that of *Aesiocopa* Zeller, 1877. The signum is long, narrow, and somewhat slender crescent-shaped, most likely homologous with that of *Aesiocopa* Zeller, 1877, *Amorbia* Clemens, 1860, *Amorbimorpha* Kruse, 2011, *Coelostathma* Clemens, 1860, *Lambertiodes* Diakonoff, 1959, *Paramorbia* Powell & Lambert, 1986, *Rhynchophyllus* Meyrick, 1932, *Sparganocosma* Brown, 2013, *Sparganopseustis* Powell & Lambert, 1986, *Sparganothina* Powell, 1986, and *Sparganothoides* Lambert & Powell, 1986. *Circanota* lacks abdominal dorsal pits, which are present in *Aesiocopa*, many species of *Amorbia*, *Coelostathma*, and *Sparganopseustis*. In the male genitalia of *Circanota*, the uncus is long and slender, as in many other sparganothine genera (e.g., *Sparganothis* Hübner, 1825, *Cenopis* Zeller, 1875) and in contrast to the spindle-shaped (i.e., broadened subbasally) uncus of *Platynota*. The secondary arms of the socii are long and slender, more similar to those of males of genera whose females lack the crescent-shape signum (e.g., *Sparganothis*, *Cenopis*, *Platynota*). The valvae of *Circanota undulata* are highly modified, whereas those of *Circanota simplex* are less so, although in both species the distal edge of the valva (the area between the termination of the sacculus and the termination of the costa) is membranous and somewhat lobed (much more pronounced in *Circanota undulata*).

#### Description.

Head: Vertex relatively smooth scaled, upper frons with large, flat tuft of scales overhanging lower frons, lower frons smooth scaled without complex hood. Antennal scaling in two bands per segment, sensory setae 0.7–0.9 times flagellomere width in male, shorter, sparser in female; labial palpus moderate in length, segment II about 2.0 times horizontal diameter of compound eye in male, only slightly longer in female, weakly upcurved; ocellus well developed in both sexes. Thorax: Notum smooth scaled throughout; legs unmodified. Forewing length 4.9–6.1 mm, slightly greater in females; costa undulate in both sexes; costal fold present in male, broad and pronounced in *undulata*, reduced and narrow in *simplex*; forewing without raised scales; R_4_ and R_5_ stalked in basal 0.6. Hindwing with Rs and M_1_ approximate at base, CuA_1_ and M_3_ connate, and M_2_ and M_3_ approximate at base; cubital hair pecten present in both sexes, slightly less developed in males. Abdomen: Dorsal pits absent. Female lacking enlarged corethrogyne scaling. Male genitalia with uncus long, slender, uniform in width throughout, curved ventrad apically; socius rather short, narrow, with slender line of sclerotization along inner edge, bearing long dense scales, secondary arm long, slender, not expanded apically; gnathos absent; transtilla slightly arched mesially, with few (*undulata*) or many (*simplex*) stout spines; pulvinus weakly developed; valva broad, short, with expanded “notch” subapically (in *undulata*); sacculus narrow, confined to basal edge of valva, either simple, without free distal process (*simplex*) or undulate with a long, free, weakly curved spine at termination (*undulata*). Phallus long, slender and curved in *undulata*, shorter, more pistol shaped in *simplex*; vesica with a field of about 25–30 short, slender, deciduous, asciculate cornuti. Female genitalia with papillae anales oblong-ovoid, slightly narrower anteriorly, densely covered with papillate setae throughout; apophyses about as long as papillae anales, posteriores slightly shorter than anteriores; sterigma a strongly sclerotized fig, flat along posterior margin, slightly rounded anteriorly, in *undulata* with a conspicuous, angulate-rhomboidal mesal portion immediately before junction with ductus bursae (typically the cup-shaped portion of the sterigma), in *simplex* simple, flat, unmodified; colliculum inconspicuous; ductus bursae uniformly narrow throughout, equal to or slightly longer than corpus bursae; corpus bursae round, entire surface with fine faint rounded punctations; signum a ribbon-like, crescent-shaped sclerite in posterior portion of corpus bursae; a tiny, membranous, pocket-like external evagination near signum.

#### Distribution and biology.

*Circanota* includes two species: *Circanota undulata* from Costa Rica and Panama, and *Circanota simplex* from Panama and Ecuador. Hence, the documented range extends from southern Central America to northern South America. *Circanota undulata* has been collected from about 50–500 m in elevation, with a single individual from 900 m; and *Circanota simplex* is known from below 600 m.

Although the early stages of *Circanota* are unknown, circumstantial evidence suggests that larvae may feed in leaf litter, as was hypothesized for the related *Sparganothoides* (Kruse and Powell 2009). *Circanota undulata* is not among the species reared during the extensive survey of the caterpillars of Area de Conservación Guanacaste in northwestern Costa Rica (Janzen and Hallwachs 2014); however, it has been collected at light (n = 8 specimens) within the same study area. Because most Sparganothini are polyphagous leaf-rollers (Powell and Brown 2012), it is assumed that larvae of this species would have been encountered if it was feeding externally on living vegetation. Although leaf-litter feeding is unusual within Tortricidae, it is the main feeding mode in the Australian Epitymbiini (Tortricinae) (Powell and Common 1985) and has been implicated as the feeding strategy in the Nearctic genus *Anopina* Obraztsov, 1962 (Tortricinae: Euliini) (Brown and Powell 2000) and the Neotropical genus *Sparganothoides* (Kruse and Powell 2009).

#### Barcodes.

BOLD (Barcode of Life Database, Biodiversity Institute of Ontario, University of Guelph) includes sequence data for 12 specimens of *Circanota undulata* but no specimens of *Circanota simplex*. Of the 12 specimens, I have examined four from the ALAS Project (The Arthropods of La Selva) (Colwell and Longino 2006) (INBio) and three from Area de Conservación Guanacaste (Janzen and Hallwachs 2014) (USNM). Five specimens from Area de Conservación Guanacaste could not be located. The 12 specimens show genetic divergence of less than 0.1% among the samples. In neighbor-joining trees (based on COI) for all Spaganothini, *Circanota* is portrayed nearest *Sparganothoides*, consistent with many morphological features (e.g., the crescent-shaped signum, the presence of secondary arms of the gnathos, the absence of dorsal pits, minimal sexual dimorphism).

#### Remarks.

The male genitalia of the two included species are divergent in several features, in particular the shapes of the valva and the phallus, casting some doubt on their putative congeneric status. However, the two species are virtually indistinguishable in facies, including the most compelling synapomorphy of the genus (i.e., undulate costa in both sexes), and the male genitalia share a unique combination of characters: a long, slender uncus; short socii with long, slender secondary arms (not expanded distally); and a membranous lobelike process at the outer margin of the valva. Both species also lack modified scaling on the frons in the male (which is present in many *Platynota*) and dorsal pits (which are present in many *Amorbia* and *Sparganopseustis* and nearly all *Coelostathma*, *Aesiocopa*, and *Sparganopseustis*). On the basis of these characters, the two species are assigned to *Circanota*.

#### Etymology.

The generic name is from the Latin “circum”, meaning around, and the Latin “nota,” meaning mark. It is interpreted as masculine.

### Circanota undulata

Taxon classificationAnimaliaLepidopteraTortricidae

Brown sp. n.

http://zoobank.org/363BF9EF-58B2-4C09-9067-9B4400939B41

**Figures 1–8.**
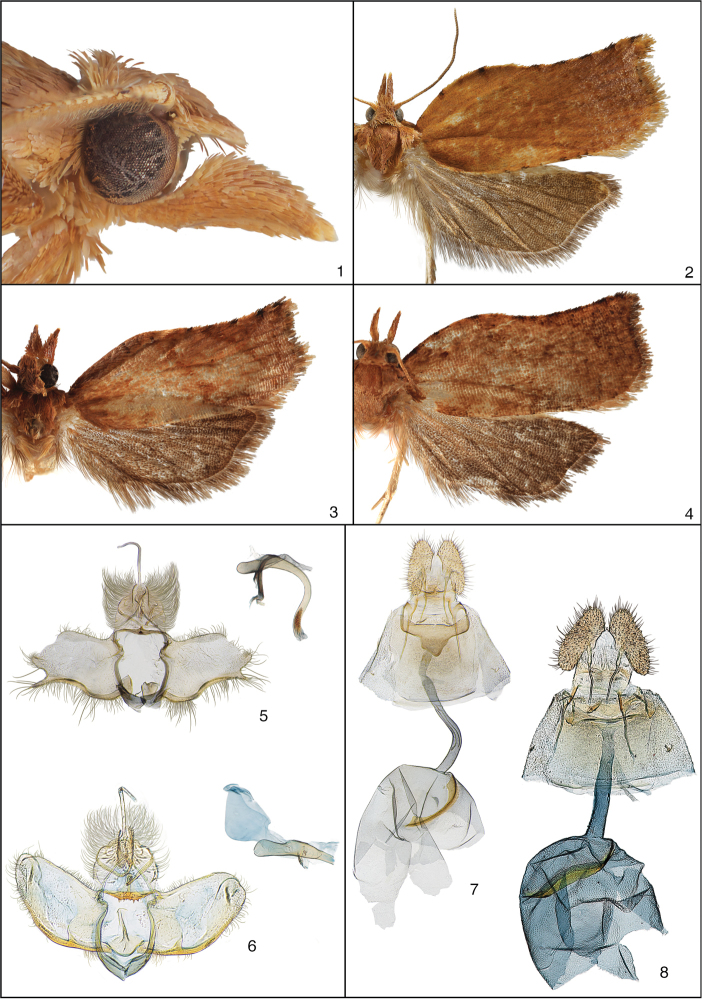
Features of the adult of *Circanota*. **1** Head of male paratype of *Circanota undulata* from Turrialba, Costa Rica **2** Adult female paratype of *Circanota undulata* from Estación Biologia La Selva, Costa Rica **3** Adult male holotype of *Circanota undulata* from Estación Biologia La Selva, Costa Rica **4** Adult male paratype of *Circanota simplex* from Tinalandia, Ecuador **5** Male genitalia of holotype of *Circanota undulata* (USNM slide 118,864) **6** Male genitalia of paratype of *Circanota simplex* from Ecuador (USNM slide 142,059) **7** Female genitalia of paratype of *Circanota undulata* from 11 km ESE La Virgen, Costa Rica (USNM slide 118,863) **8** Female of paratype of *Circanota simplex* from Barro Colorado Island, Panama (USNM slide 144,903).

Figs 1 , 2 , 3 , 5 , 7

#### Diagnosis.

In *Circanota undulata* the valvae are short and broad, the membranous distal edge of the valva between the termination of the costa and the median lobe of the outer margin is exceedingly long, and the shape of the sacculus is unique - long and undulate, ending in a slender free spinelike process. All of these features are in strong contrast to their condition in *Circanota simplex*, which has a rounded valva that is bilobed disally, and a short, narrow sacculus lacking a free distal tip. The female genitalia of *Circanota undulata* have an asymmetrical anterior projection of the sterigma (= antrum) that is lacking in *Circanota simplex*.

#### Description.

Male. Head: Vertex and upper frons uniform fawn brown, lower frons pale cream. Labial palpus fawn brown, paler on inner surface. Antenna pale fawn brown, slightly darker on scape. Thorax: Tegula and notum fawn brown. Forewing length 4.9–5.5 mm (mean 5.1; n = 6), fawn brown mixed throughout with pale orange brown, with faint, narrow, variable traces of slightly darker post-median and subterminal faciae, and a few short darker strigulae along costa; well developed costal fold occupying straight basal 0.4 of costa. Hindwing uniform dark gray brown. Abdomen: Genitalia (Fig. 5) with uncus long, slender, uniform in width throughout, hooked ventrad in apical 0.25; socius rather short, narrow, with slender line of sclerotization along inner edge, bearing long dense scales, secondary arm long, slender, not expanded apically; transtilla weakly expanded mesially with a single small median spine; valva broad, short, with costa short, well defined; an irregular “notch” extending from distal end of costa to lobe-like process near middle of outer margin of valva; sacculus well-defined, confined to basal edge of valva, undulate with a long, free, weakly curved spine at termination, a rounded excavation between tip of sacculus and lobe at middle of outer margin of valva. Phallus long, slender, undulate, nearly uniform in width throughout, with phallobase slightly expanded; vesica with a field of about 25–30 short, slender, deciduous (based on presence in female ductus bursae), asciculate cornuti.

Female. Head and Thorax: Essentially as described for male, except forewing length 5.0–5.9 mm (mean 5.5; n = 6) and forewing slightly darker overall. Abdomen: Genitalia (Fig. 7) as described for genus; sterigma a sclerotized fig, flat along posterior margin, slightly rounded anteriorly, confluent with an angulate-rhomboidal mesal portion at junction of sterigma and ductus bursae (typically the cup-shaped portion of the sterigma); ductus bursae uniformly narrow throughout, slightly longer than corpus bursae; ductus seminalis arising from ductus bursae ca. 0.33 distance from ostium to junction with corpus bursae; corpus bursae round, signum a ribbon-like, crescent-shaped sclerite, nearly uniform in width.

Holotype. Male, Costa Rica, Heredia, Estación Biologia La Selva, 50–150 m, 10°26'N, 84°01'W, 12 May 1999, INBio-OET [BOLD: TONA407-08; JWB-08-0133]. Deposited in INBio.

Paratypes (11♂, 11♀). COSTA RICA: Alajuela: Area de Conservación, 09-SRNP-101007 (1♂) [BOLD: BLPDF403-09], 10-SRNP-106423 (1♂) [BOLD: BLPDR798-10], 10-SRNP-106757 (1♀) [BOLD: BLPDS133-10], D. Janzen & W. Hallwachs (USNM). Cartago: Turrialba, 17–21 Feb 1965 (1♂, 1♀), 13–17 Mar 1965 (1♂), S. S. & W. D. Duckworth (USNM). Heredia: Estación Biologia La Selva, 50–150 m, 10°26'N, 84°01'W, 10 Jan 1993 (1♀), 12 Mar 1998 (1♀), 14 May 1998 (1♀), 21 Apr 1999 (1♂), INBio-OET, 26 Feb 2003 (1♂), 23–29 Feb 2004 (1♀) [BOLD: TONA408-08; JWB-08-0134], D. Wagner (INBio). 11 km ESE La Virgen, 250–350 m, 10°21'N, 84°03'W, 16 Mar 2004 (1♂), 18 Mar 2004 (1♂), 20 Mar 2004 (1♀), 21 Mar 2004 (1♂), 7 Apr 2004 (1♀) [BOLD: TONA409-08; JWB-08-0135], INBio-OET-ALAS transect (INBio). 10 km SE La Virgen, El Ciebo Ranger Station, 450–550 m, 10°20'N, 84°05'W, 11–12 Feb 2003 (1♂) [BOLD: TONA406-08; JWB-08-0132], D. Wagner (INBio), 17–23 Mar 2003 (1♀), 21 May 2003 (1♂), INBio-OET (INBio). Puntarenas: Golfito, 25–28 Apr 1965 (1♀), S. S. & W. D. Duckworth (USNM). PAMANA: Cabima, May 1911 (1♂), A. Busck (USNM). Cocle, El Valle, 800–900 m, 3–5 Jan 1988 (1♀), MacDonald & Schiefer (MEM).

#### Barcodes.

In neighbor-joining trees, barcode sequence data from *Circanota undulata* (n = 12 field collected adults) form a tight cluster with genetic divergence of less than 0.1% among the individuals.

#### Distribution and biology.

*Circanota undulata* is recorded from Costa Rica and Panama below about 900 m elevation. All the specimens were collected between January and May.

#### Etymology.

The specific epithet refers to the undulate costa of the forewing.

### Circanota simplex

Taxon classificationAnimaliaLepidopteraTortricidae

Brown sp. n.

http://zoobank.org/1DDED0AC-8C88-4D62-BB55-261003E92F35

Figs 4 , 6 , 8

#### Diagnosis.

*Circanota simplex* can be distinguished superficially from *Circanota undulata* by the much narrower costal fold of the male forewing, which is broad and well defined in the latter. Otherwise, the two species are indistinguishable. In contrast, the male genitalia are extremely dissimilar between the two: those of *Circanota simplex* are much less modified than those of *Circanota undulata*, with a simple sacculus, a more elongate-rounded, somewhat bilobed valva, and a phallus that is much shorter and more pistol shaped. The female genitalia of *Circanota simplex* likewise are dissimilar to those of *Circanota undulata*, with a symmetrical sterigma compared to the asymmetrical anterior extension of the sterigma (= antrum) in *Circanota undulata*.

#### Description.

Male. Head: Vertex and upper frons uniform fawn brown, lower frons pale cream. Labial palpus fawn brown, paler on inner surface. Antenna pale fawn brown, slightly darker on scape. Thorax: Tegula and notum fawn brown. Forewing (Fig. 4) length 6.0 mm (n = 1); fawn brown mixed throughout with pale orange brown, with faint, narrow, variable traces of slightly darker post-median and subterminal facia, and a few short darker markings along costa; male with costal fold weakly developed, occupying straight basal 0.4 of costa. Hindwing uniform dark gray brown. Abdomen: Genitalia (Fig. 6) with uncus long, slender, uniform in width throughout, curved in distal 0.2; socius rather short, narrow, with slender line of sclerotization along inner edge, bearing long dense scales, secondary arm long, slender, not expanded apically; transtilla slightly arched mesially, with several stout spines; valva short, broad, bilobed rounded distally; sacculus narrow, simple, confined to basal edge of valva, lacking free distal process. Phallus short, pistol shaped; vesica with a field of about 25–30 aciculate, presumably deciduous cornuti.

Female: Head and Thorax: Essentially as described for male, except forewing length 7.0–8.0 mm (mean 7.5; n = 3) and forewing with pattern elements less defined. Abdomen: Genitalia (Fig. 8) with sterigma a narrow sclerotized fig; colliculum weakly developed; ductus bursae uniformly narrow throughout, about as long as corpus bursae; ductus seminalis arising from ductus bursae ca. 0.2 length from ostium to junction with corpus bursae; corpus bursae round, signum weakly curved, ribbon-like.

Holotype. Male, Panama, [Canal Zone], Barro Colorado Island, 1–9 May 1964, W. D. & S. S. Duckworth. Deposited in USNM.

Paratypes (1♂, 3♀). PANAMA: Canal Zone: Barro Colorado Island, 1–9 May 1964 (3♀), W. D. & S. S. Duckworth (USNM). ECUADOR: Pichincha, Tinalandia, 16 km E Santo Domingo de los Colorados, 600 m, 5–11 May 1990 (1♂), R. H. Leuschner (USNM).

#### Distribution and biology.

*Circanota simplex* is known from Panama and Ecuador, from about sea level to 600 m elevation. Specimens have been collected only in May, but that likely reflects sampling bias rather than a narrow flight period.

#### Etymology.

The specific epithet refers to the simple, unmodified features of the genitalia as compared with those of *Circanota undulata*.

#### Remarks.

The single male from Ecuador agrees well with the holotype of *Circanota simplex*, but the ventral lobe of the valva is slightly broader in the former. Until additional evidence becomes available, this slight difference is assumed to represent geographic variation.

## Discussion

The slender crescent-shaped signum of the female genitalia of *Circanota* represents a putative synapomorphy for a sparganothine clade that includes *Aesiocopa*, *Amorbia*, *Amorbimorpha*, *Circanota*, *Coelostathma*, *Lambertiodes*, *Paramorbia*, *Rhynchophyllus*, *Sparganocosma*, *Sparganopseustis*, *Sparganothina*, and *Sparganothoides* (Brown et al. 2013, Brown 2014). Within this clade, *Circanota* shares the presence of secondary arms of the socii with *Aesiocopa*, *Amorbimorpha*, *Sparganopseustis*, and *Sparganothoides*, but the arms are much more slender throughout in *Circanota*. In neighbor-joining trees that include all of the BOLD data for Sparganothini (Brown et al. in preparation), *Circanota* is portrayed nearest *Sparganothoides*, and *Aesiocopa* nearest *Sparganopseustis*; there are no sequence data for *Amorbimorpha*. *Circanota* is one of five genera (i.e., *Aesiocopa*, *Amorbimorpha*, *Circanota*, *Sparganopseustis*, and *Sparganothoides*) that possess both a crescent-shaped signum and secondary arms of the gnathos. Among these genera, *Circanota* shares the absence of dorsal pits and minimal sexual dimorphism with *Amorbimorpha* and *Sparganothoides*. All described *Amorbimorpha* and many *Spaganothoides* have a distally bifurcate uncus, remarkably different from the slender, curved uncus of *Circanota*. Hence, although many features suggest a strong affinity between *Sparganothoides* and *Circanota*, the two genera are divergent based on several conspicuous features of the adult morphology.

## Supplementary Material

XML Treatment for Circanota

XML Treatment for Circanota undulata

XML Treatment for Circanota simplex
